# A Chemical Structure and Machine Learning Approach to Assess the Potential Bioactivity of Endogenous Metabolites and Their Association with Early Childhood Systemic Inflammation

**DOI:** 10.3390/metabo14050278

**Published:** 2024-05-10

**Authors:** Mario Lovrić, Tingting Wang, Mads Rønnow Staffe, Iva Šunić, Kristina Časni, Jessica Lasky-Su, Bo Chawes, Morten Arendt Rasmussen

**Affiliations:** 1 COPSAC, Copenhagen Prospective Studies on Asthma in Childhood, Herlev and Gentofte Hospital, 2820 Gentofte, Denmark; 2 Centre for Applied Bioanthropology, Institute for Anthropological Research, 10000 Zagreb, Croatia; iva.sunic@inantro.hr; 3 The Lisbon Council, 1040 Brussels, Belgium; 4 Department of Food Science, University of Copenhagen, 1958 Frederiksberg, Denmark; 5 Know-Center, 8010 Graz, Austria; 6 Department of Medicine, Boston, MA 02115, USA; 7 Channing Division of Network Medicine, Brigham and Women’s Hospital, Harvard Medical School, Boston, MA 02115, USA; 8 Department of Clinical Medicine, Faculty of Health and Medical Sciences, University of Copenhagen, 2300 Copenhagen, Denmark

**Keywords:** metabolomics, QSAR, inflammation, cortisol, cortisone, vitamin A, CRP

## Abstract

Metabolomics has gained much attention due to its potential to reveal molecular disease mechanisms and present viable biomarkers. This work uses a panel of untargeted serum metabolomes from 602 children from the COPSAC2010 mother–child cohort. The annotated part of the metabolome consists of 517 chemical compounds curated using automated procedures. We created a filtering method for the quantified metabolites using predicted quantitative structure–bioactivity relationships for the Tox21 database on nuclear receptors and stress response in cell lines. The metabolites measured in the children’s serums are predicted to affect specific targeted models, known for their significance in inflammation, immune function, and health outcomes. The targets from Tox21 have been used as targets with quantitative structure–activity relationships (QSARs). They were trained for ~7000 structures, saved as models, and then applied to the annotated metabolites to predict their potential bioactivities. The models were selected based on strict accuracy criteria surpassing random effects. After application, 52 metabolites showed potential bioactivity based on structural similarity with known active compounds from the Tox21 set. The filtered compounds were subsequently used and weighted by their bioactive potential to show an association with early childhood hs-CRP levels at six months in a linear model supporting a physiological adverse effect on systemic low-grade inflammation.

## 1. Introduction

Metabolomics aims to capture a broad range of small molecules, either quantitatively by measuring their concentration or qualitatively by identifying their presence and structure, which are essential intermediates and end products of metabolism in organisms. One can analyze pathological processes underlying a disease or physiological state of interest using rich metabolomics data, e.g., obtaining biomarkers [1] or predicting responses to therapy [2,3]. Furthermore, one can use the metabolome for phenotyping organisms or diseases [4]. With such methods, one can also delve into a mechanistic information layer for understanding modes of action regarding exposures such as diet, exercise, and pollutants on disease progression [5]. Metabolomic instruments provide rapid, cost-effective, and sensitive results from many possible biological samples. Yet, with an appropriate analysis, the data they produce are meaningful. It is essential to interpret these data to build biochemical pathways and comprehend their interactions in healthy and diseased conditions [6]. Liquid chromatography–mass spectrometry (LC-MS), one of the most commonly used approaches for metabolomics studies, can achieve a high number of annotations [7], which was also essential to this study.

### 1.1. hs-CRP as a Target for Physiological Conditions

Another significant predictor of health is the assessment of levels of C-reactive protein with high-sensitivity methods (hs-CRP), which is generally considered a marker of systemic low-grade inflammation. Elevated hs-CRP has been shown in early-onset diseases and has been associated with an increased prevalence of overweight and obesity in children [8,9]. Further, studies have shown that elevated hs-CRP is a biomarker of childhood asthma [10] and allergy [11] but is also associated with Attention deficit hyperactivity disorder (ADHD) development [12]. Hence, hs-CRP is a valuable tool for understanding childhood obesity risk, asthma, allergy, and overall health status. Elevated hs-CRP is hence interpreted as a physiological state of systemic low-grade inflammation in this study. The expectation is, therefore, that inflammation is related to metabolic processes and should hence be reflected in the metabolome by, e.g., other biomarkers like the previously presented GlycA [13].

### 1.2. Signals in the Metabolome

The leading assumption in this work is that one can use chemical information of endogenous or exogenous metabolites to relate them to their biological activity and consequently with physiological conditions. This can be processed using quantitative structure–activity relationships (QSARs). QSARs are based on mapping a feature space (X), calculated from chemical structures, on a target chemical or biological activity (y) [14,15]. Hence, given a chemical structure, one should be able to understand the compound’s biological activity, a concept relevant in drug discovery and toxicology. In reverse, after developing a QSAR, one can predict biological activity for a biological target (e.g., an enzyme or receptor) for a new chemical structure, given some chemical similarity to previously utilized chemical space. To achieve this with metabolites, one must know their structures. Herein, the biological activities of interest are the activation or inhibition of specific biochemical pathways from the Tox21 listed targets [16,17]. The selected Tox21 data set for modeling includes 12 bioactivity endpoints, 5 related to stress response (SR) and 7 to nuclear receptor (NR) panels. NRs are a class of transcription factors involved in regulating gene expression. Once the models are trained, and the relationship of chemical structure and bioactivity is set, they can be used to assess bioactivities of other chemical structures, i.e., metabolites.

### 1.3. Aim of This Work

This work aims to filter metabolites from many compounds to inspect associations with potential biological activities and hence with physiological conditions. Filtering is based on structural similarities of metabolites to known bioactive compounds against the Tox21 targets, which can be potentially bioactive in unspecified pathways. Hence, dysregulated, endogenous compounds could be associated with physiological states. Even though there is not much research on the endobiotic effects and associations, there are indications for such. Elevated cortisol concentrations have been associated with psychiatric diseases [18,19]. Other metabolites like phenylalanine are known toxicants in conditions such as phenylketonuria [20]. Research also suggests that toxic intermediates in metabolomic pathways [21] can contain reactive functional groups and be leveraged, e.g., for cancer therapy. A set of QSAR models is developed in this work against the Tox21 biological target and used to predict potential outcomes for each of the quantified metabolites. The expected outcomes are then used as filters to select potentially bioactive compounds that could trigger the given Tox21 pathways given their chemical structure. The filtered metabolites were evaluated based on their abundance and bioactivity against levels of hs-CRP in the children at six months of age.

## 2. Materials and Methods

### 2.1. Cohort, Metabolomics Profiling, and hs-CRP Assessment

#### 2.1.1. Cohort

**Figure 1:**
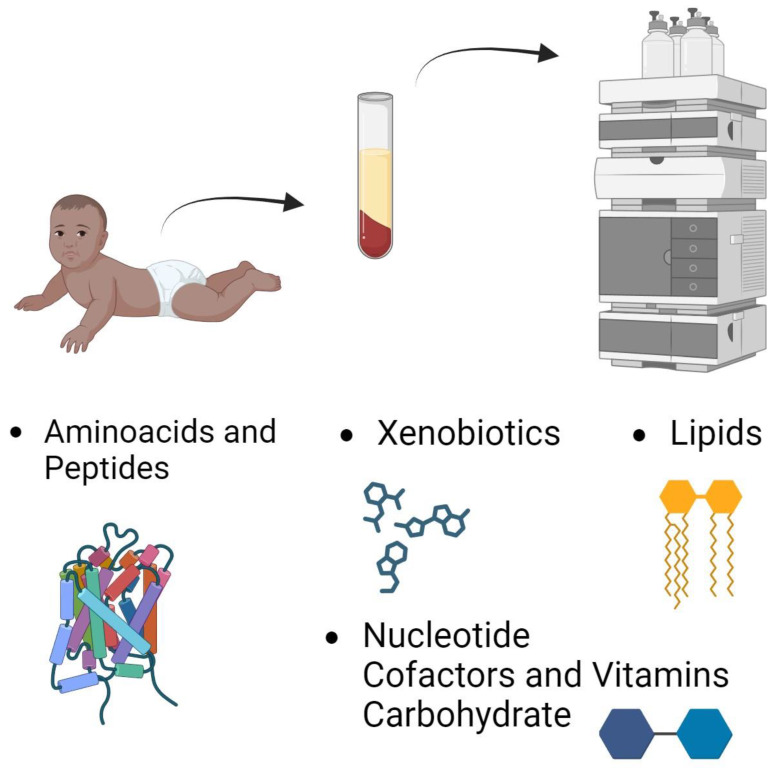
Metabolome sampling and chemical categories in the COPSAC2010 cohort selected for this work.

The COPSAC2010 cohort comprises a non-selected group of mother–child pairs, including 738 pregnant women and their 700 children. The women were recruited between gestational weeks 22 and 26 during their first pregnancy examinations. Of the 738 pregnant women (with an average age of 32.3 ± 4.3 years at the time of their child’s birth), 700 children were enrolled in the study. Since then, the children have been visiting the clinic regularly. Gestational age was determined using routine pregnancy care ultrasonography. The COPSAC2010 study participants included infants delivered pre-term and post-term (30–42 weeks). During the third trimester, the women took part in a double-blind, randomized controlled trial with a factorial design, receiving either high-dose (2800 IU/day) or standard-dose (400 IU/day) [22] vitamin D and either 2.4 g n-3 long-chain polyunsaturated fatty acid (LCPUFA, 55% (*w*/*w*) 20:5 (n-3) eicosapentaenoic acid (EPA) and 37% (*w*/*w*) 22:6 (n-3) docosahexaenoic acid (DHA)) or placebo (72% (*w*/*w*) n-9 oleic acid and 12% (*w*/*w*) n-9 linoleic acid) [23]. Women with endocrine, heart, or kidney diseases or a daily vitamin D intake above 600 IU/day were excluded. Children with a gestational age of less than 32 weeks were also excluded. The trial received approval from the National Committee on Health Research Ethics (H-B-2008-093) and the Danish Data Protection Agency (2015-41-3696). Both parents provided oral and written informed consent before enrolment. The full protocol for the recruitment, information on withdrawals from the study, and a flowchart of the study were previously presented in the Supplementary Materials (Protocol and Supplementary Appendix) of this group’s previous work [23]. Figure 1 depicts the metabolomic part of this study.

#### 2.1.2. Blood Collection and Metabolite Quantification

Blood samples were taken from children at the age of six months. Blood was collected in an ethylenediaminetetraacetic acid (EDTA) tube and left at room temperature for 30 min before being centrifuged for 10 min at 4000 rpm. The supernatant was collected and stored at −80 °C for future analysis [24]. The methods for sample preparation, UHPLC-MS/MS analysis, and quality control are comprehensively described in reference [25]. In this study, an untargeted metabolomic analysis of plasma from mothers and their children was conducted at Metabolon, Inc. using a Waters ACQUITY UHPLC (Milford, MA, USA) and a ThermoFisher Scientific (Waltham, MA, USA) QExactive™ Hybrid Quadrupole-Orbitrap™ mass spectrometer with a heated electrospray ionization source, operating at a resolution of 35,000 mass units. The processed samples underwent analysis on four specialized platforms tailored for different classes of compounds: UHPLC-ESI(+)MS/MS for hydrophilic compounds, UHPLC-ESI(+)MS/MS for hydrophobic compounds, reverse-phase UHPLC-ESI(−)MS/MS optimized for basic conditions, and HILIC/UHPLC-ESI(−)MS/MS. The identification of metabolites was based on retention time or index range, a mass accuracy within ±10 ppm, and MS/MS spectra. Compound identification was based on the following criteria: (1) compounds labeled with “*” have identification level 2; (2) compounds labeled with “**” have level 3 (since no standards or matching spectra are available); (3) compounds named with “X-” are unknown and therefore have level 4; and (4) if no label is applied, the identification level is 1 [26]. The analyses were conducted in Metabolon, Inc. in Morrisville, NC, USA. Additional details on the analysis were previously published in [27].

#### 2.1.3. Assessment of hs-CRP Levels

Children at the age of six months had blood drawn from a cubital vein into an EDTA tube. The samples were centrifuged to separate plasma and cells and stored at −80 °C until analysis. After the samples were thawed, the hs-CRP levels were measured using a high-sensitivity electrochemiluminescence-based assay from Meso Scale Discovery. Duplicate measurements were taken and analyzed using the Sector Image 2400 A from Meso Scale Discovery in Gaithersburg, MD. The lower limit of detection for CRP was 0.007 ng/mL [28].

### 2.2. Data Preparation and Model Building

#### 2.2.1. Data Preparation for Metabolites

The metabolites with more than 33% missing values (67% values per feature present) were removed from the analysis, referring to our previous works to keep methodological consistency. Some literature sources recommend imputing features above 70% [29], which is close to the threshold in this work. The variables that passed this threshold, i.e., with less than 33% missing values, were then imputed with 1/10 of the minimum concentration per metabolite (under the assumption to correspond to the detection limit). The metabolites were then scaled to 0–1 (min–max scaling). A further cleaning step was the removal of low-variance metabolites, i.e., the lowest 10% of metabolites by variance [30]. The last step was removing highly correlated metabolites with above 90% Pearson correlation. Finally, 517 compounds were successfully converted to simplified molecular-input line-entry system (SMILES) encodings of molecules, resulting in a final data set for the analysis.

#### 2.2.2. Bioactivity Assessment Pipeline

**Figure 2:**
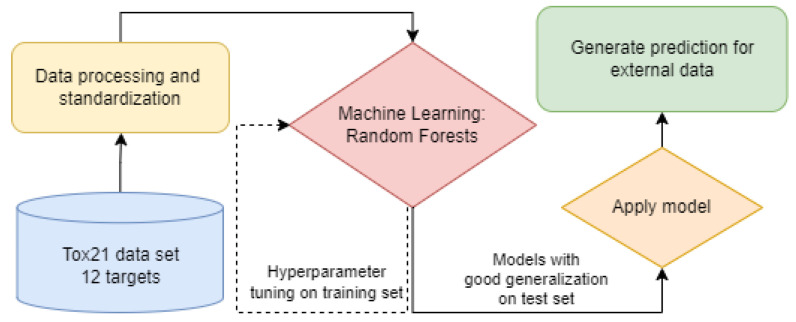
The modeling pipeline in this study.

The model training and application pipeline is presented in Figure 2. The pipeline starts with data extraction and preparation for building QSAR models (blue and yellow rectangles in the figures), described in Section 2.2.2 and Section 2.2.3. Once the data are prepared, models are built using Random Forests and hyperparameter optimization (Section 2.2.4) on features generated from chemical structures. These models can predict bioactivity against biological targets such as the 12 given in Section 2.2.2. Once the models are generated and validated, the final step is to predict bioactivities for the 12 targets per quantified metabolite and patient. The predictions then enter further statistical analysis to reveal an association with hs-CRP levels.

#### 2.2.3. Bioactivity Data

The chosen bioactivities for this task stem from the Tox21 compound library [16,17] provided by the U.S. Environmental Protection Agency (EPA). The Tox21 program, which was also part of the Tox21 challenge [16], is also where data can be downloaded from; this includes data on the androgen receptor (AR), estrogen receptor (ER), progesterone receptor (PR), aromatase receptor, peroxisome proliferator-activated receptor gamma (PPAR), and the aryl hydrocarbon receptor (AhR). The compounds in the library were tested against their ability to interact with various SR pathways, including antioxidant-responsive elements (ARE), p53 tumor proteins, mitochondrial membrane potential (MMP), proteins involved in DNA damage repair (ATAD5), and heat shock factor response elements (HSE). Numerous studies have been conducted on this data set, and the outcomes have been reported in various reports [31,32,33,34]. Hence, it represents a baseline data set for building bioactivity QSARs since it is imbalanced, chemically diverse, and one of the more significant publicly available data sets (~10 k compounds). Because of the challenges presented by this data set, it has been extensively studied in cheminformatics research in machine learning methods [31,34], class balancing methods [33,35], the testing of different chemical representations [36], and multitasking/deep learning [34]. The raw data were pre-processed, as they contain duplicate structures that consider the active or organic part of the molecules. This was also reported earlier [33]. Molecules with invalid structural identifiers were removed, and those that were valid were converted to their canonical SMILES [37]. Duplicates were either removed by IDs or SMILES. Our methodology employed a comprehensive standardization protocol to ensure the uniformity and accuracy of chemical structure representations before the computational analysis. The structure preparation pipeline inspired by procedures [33,38] and implemented in the ChemAxon Standardizer (v18.28.0, ChemAxon, Budapest, Hungary) tool is represented in a defined manner in the <StandardizerConfiguration> XML schema (https://zenodo.org/records/10888738, accessed on 25 April 2024).The protocol consists of following steps: (1) Removal of Explicit Hydrogens; (2) Dearomatization; (3) Conversion of Pi-metal Bonds; (4) Disconnection of Metal Atoms; (5) Stripping of Salts; (6) Fragment Removal; (7) Transformation of Diazonium Groups; (8) Neutralization; (9) Wedge Clean; (10) Mesomerization and Tautomerization; (11) Aromatization; (12) 2D Cleaning. Once the structures were ready, the molecular descriptors Morgan fingerprints (FPR) [39], MACC keys, and 200 molecular descriptors were calculated for the 8314 structures using the RDKit library [40]. To reduce possible bit collision in the fingerprints [41,42], the fingerprint vectors were set to 5120 bits and a radius of 2. The Python scripts used for this work were previously published [43]. The data sets are available in the Supplementary Materials (https://zenodo.org/records/10888738, accessed on 25 April 2024). In the final prepared data sets, the SR-HSE activator (PubChem AID: 743228) assay recorded 6402 observations with 340 positives, while the NR-AR agonist assay observed 7060 instances, identifying 306 positives. The SR-ARE agonist (AID: 743219) assay demonstrated a higher activity with 945 positives out of 5758 observations. The NR-Aromatase antagonist (AID: 743139) and NR-ER-LBD (AID: 743077) assays reported 304 and 343 positives from 5652 and 6817 observations, respectively. Notably, the NR-AhR agonist (AID: 743122) assay detected 756 positives among 6459 observations, and the SR-MMP disruptor (AID: 720637) assay found 903 positives in 5715 cases. The NR-ER agonist assay revealed 806 positives from 6056 observations, and the NR-PPAR-gamma assay agonist (AID: 743140) had 197 positives out of 6355. The SR-p53 agonist (AID: 720552) assay identified 423 positives among 6643 observations, the SR-ATAD5 inducer (AID: 720516) assay found 275 positives in 6926 cases, and the NR-AR-LBD agonist (AID: 743053) assay reported 234 positives from 6617 observations.

#### 2.2.4. Machine Learning Models

Machine learning models were trained using Python (www.python.org, v3.9.1.) and its library sci-kit-learn [44] based on previous works [36,43]. Due to class imbalance in the Tox21 set, model penalization and optimization techniques were used to improve classification quality. Before model training, the data with the 12 bioactivity endpoints were split into two random subsets of 80% and 20% per endpoint individually. The penalty in scoring during hyperparameter optimization was based on the Matthews Correlation Coefficient (MCC) [45,46] defined by Equation (1), where TP (True Positive), TN (True Negative), FN (False Negative), and FP (False Positive) are the elements of the confusion matrix (https://en.wikipedia.org/wiki/Confusion_matrix, accessed on 25 April 2024). Another important metric used in this work is Balanced Accuracy, shown in Equation (2). A critical assessment of metrics for imbalanced classification QSAR models was given in previous works [46,47].

(1) $$MCC=\frac{TP\times TN-FP\times FN}{\sqrt{\left(TP+FP\right)\times \left(TP+FN\right)\times \left(TN+FP\right)\times \left(TN+FN\right)}}$$

(2) $$Balanced\;Accuracy=\frac{1}{2}\;\left(\frac{TP}{TP+FN}+\frac{TN}{TN+FP}\right)\;$$

Bayesian hyperparameter optimization (BO) was used for hyperparameter optimization [46,48] through a 10-fold cross-validation (CV) and penalizing the procedure by the MCC then iterating it 20 times and returning a set of “optimal” parameters. Hence, the MCC CV value was calculated as an average of outer folds during CV. Once the CV optimized model was generated, the model was applied on the train set each time, and for each metric, a “Train” value was generated. One of the essential model parameters was “class weight”, which contributed to classifying this imbalanced data set. During model training on the train set, feature selection using permutation importance was applied in the following steps in previous work [46]. The final model’s test set was evaluated for each target, resulting in each metric in a “Test value”.

### 2.3. Association Testing and Toxic Unit Approach

To evaluate the association with physiological conditions, the toxic unit (TU) approach was utilized [49]. TU is defined as the ratio of chemical compound concentration (ci) and the selected exposure-based toxicity value (e.g., LC_50_). Herein, a new approach is derived, namely the bioactive potential (BiP) in Equation (3). BiP is hence a product of the metabolite’s concentration (i) (relative peak area) and the probability of it being active (0–1) against a target (t). The higher the concentration and the likelihood of being bioactive, the more potent we assume the compound to be against the given targets.

(3) $${\mathrm{BiP}}_{i,t}=c_{i}\;\times \;p_{t}$$

For each child (c) in the cohort, BiP was calculated per metabolite and target and then summed and log-transformed sBIP using Equation (4), where t is the number of targets, yielding one value per child.

(4) $${sBiP}_{c,i}={log}_{10}(\sum_{t=1}^{T}{\mathrm{BiP}}_{i,t})$$

The associations were inspected by means of linear regression using the pingouin library for Python [50].

## 3. Results

### 3.1. QSAR Model Results

Eight out of twelve models yielded results above random classification (MCC CV and MCC Test > 0.3), namely for the following targets: SR-ATAD5, NR-AR-LBD, NR-AR, NR-ER-LBD, NR-ER, SR-ARE, NR-AhR, and SR-MMP (marked with an asterisk in Table 1). The threshold of 0.30 is an arbitrary choice to be in the MCC “fair agreement” regime (>0.20). Further, it reduces the risk of having imbalanced classifiers [46] while retaining a reasonable number of models. These models were then selected for further processing. While the algorithm trained for both fingerprints and descriptor sets separately, each time, the descriptors yielded better results in this setting over fingerprints with 13–38 descriptors per model (Table 1). The complete results of the models are given in the Supplementary Materials [51].

**Figure 3:**
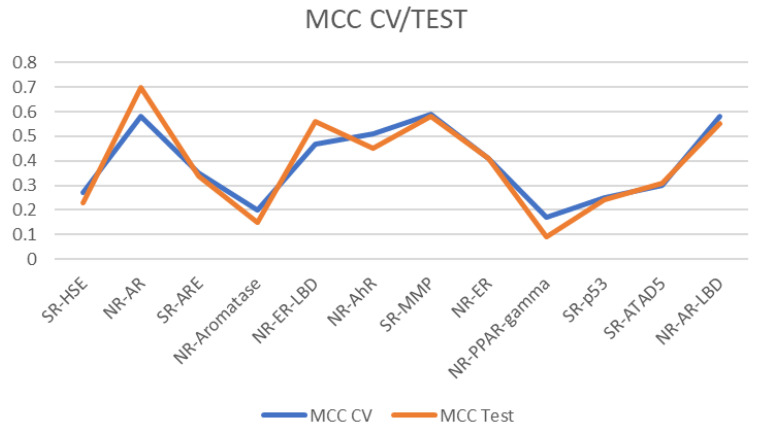
MCC CV and test set results for the classification models in Table 1.

Based on the MCC results, one can observe (Figure 3) that each model’s CV and test set assessments are well aligned and do not overfit. Our previous study on the Tox21 data set showed that these model results are aligned with models known in the literature [44], as observable in the Supplementary Materials of the cited work [44].

### 3.2. Predicting Metabolite Target Activity

The selected eight models were saved to persistent storage and applied to yield bioactivity for the 517 annotated metabolites. This resulted in a table of 517 metabolites x eight columns, namely SR-ATAD5, NR-AR-LBD, NR-AR, NR-ER-LBD, NR-ER, SR-ARE, NR-AhR, and SR-MMP. A snippet of the results is given in Table 2 to ease understanding of the model results. The results in the table are presented as probabilities instead of binary results (bioactive or not), where the convention is that a probability above 0.5 (*p* > 0.5) is considered bioactive. One can observe from the table that the probabilities show a broad range across targets. Those marked with an asterisk would be deemed bioactive towards the target since their values are above 0.5.

A mask was applied to filter out all “nonactive” results with values below 0.5 in the full results. In total, 51 metabolites showed at least one probability in one model being active above 0.5. A complete list of active metabolites is given in Table 3.

### 3.3. Association with hs-CRP

For each child (*c*) in the cohort, BiP was calculated per metabolite and target and then summed and log-transformed sBIP using Equation (2). Once a table was obtained with *sBiP* per child and metabolite, the potentially bioactive part of the metabolome was associated with levels of hs-CRP. The table was scaled priorly, and metabolites were decorrelated, reducing their number from 51 to 40. The hs-CRP levels were logarithmed, and missing values were imputed by median values. The associations were then evaluated in a linear regression. Metabolites amongst the 40 metabolites with an association with hs-CRP filtered for a regression coefficient (estimate) with a *p*-value below 0.001, chosen due to a high number of predictors to avoid false discovery, are shown in Table 4. The results show negative significant coefficients for cortisone, retinol, and 3beta-hydroxy-5-cholestenoate, while the cortisol and glycocholenate sulfate resulted in positive coefficients.

## 4. Discussion

hs-CRP is a well-known biomarker in clinical medicine and diagnostics, serving as a powerful tool for assessing low-grade or chronic systemic inflammation [52,53]. We have previously shown that systemic low-grade inflammation assessed by hs-CRP levels in pregnant mothers and their children are correlated independently of anthropometrics and environmental exposures [28]. Elevated levels of hs-CRP are associated with an increased risk of cardiovascular disease [54], inflammatory bowel disease (IBD) [55], depression [56], ADHD [12], decreased lung function in childhood [57], allergic sensitization [11], the composition of the early-life airway microbiota [58], and the risk of childhood asthma [59]. Metabolites serve as the ultimate effectors of cellular processes and represent the penultimate step in the progression to the phenotype. This underscores the importance of investigating the association between CRP and metabolites to understand better how systemic low-grade inflammation is reflected in the metabolome.

In our research, we developed a series of QSAR models targeting the Tox21 cellular endpoints. These models were employed to forecast potential outcomes against such targets for each of the quantified metabolites within the COPSAC2010 cohort. The selection of these specific metabolites was based on their abundance and bioactivity in relation to high-sensitivity C-reactive protein (hs-CRP) levels in children at six months of age. First and foremost, the model we developed has achieved reasonable generalization, as indicated by the prediction results. The Balanced Accuracy of our models for the selected eight endpoints consistently exceeded 0.6 in both the training and test sets, and the Matthews Correlation Coefficients (MCC) consistently exceeded 0.3. These metrics demonstrate the robustness of our models for making accurate predictions in the following sections. Comparing the results here with those of others in the literature is difficult since the data might have been processed differently, and the train test set can be different, too. A comparison with [60], who performed thorough modeling of the same set, results in the following observations. For the SR-ATAD5 assay, our model achieved an MCC score of 0.31, improving the literature values of 0.272 and 0.395 under different criteria. In the case of NR-AR, our model significantly outperformed the literature-reported scores for the related assay with our score of 0.7 compared to 0.481 and 0.357. The SR-ARE assay saw our model achieving a 0.34 MCC score, which is competitive with the literature values of 0.317 and 0.461. For NR-Aromatase, our model’s score of 0.15 was compared against the reported literature scores of 0.186 and 0.429. Our NR-ER-LBD model’s performance, with an MCC score of 0.56, was robust against the literature scores of 0.457 and 0.362. Similarly, for NR-AhR, our model achieved a 0.45 MCC score, slightly below the literature value of 0.513 and closely matching the second criterion score of 0.359. The SR-MMP assay model demonstrated a strong performance with an MCC score of 0.58, comparing favorably against the literature scores of 0.501 and 0.475. In the NR-ER assay, our model’s 0.41 MCC score was evaluated against literature scores of 0.235 and 0.555. For the NR-PPAR-Gamma assay, our model scored 0.09, which was weaker than the literature scores of 0.238 and 0.457. The SR-p53 assay model yielded a 0.24 MCC score compared to the literature scores of 0.356 and 0.458. Our NR-AR-LBD model’s MCC score of 0.55 was robust compared to the literature scores of 0.481 and 0.357. The results appear in similar ranges while not using the same test set and processing steps. A similar comparison of our previous models with the literature was presented in the Supplements of [46], yielding similar conclusions.

Using the developed models in this work, we identified 40 out of the 517 metabolites in our cohort that showed bioactivity in at least one of the eight models. We tested the *BiP* of these 40 metabolites in a linear model against the levels of hs-CRP in children at six months of age. We identified five metabolites of particular interest, each demonstrating regression coefficients (estimates) with a *p*-value below 0.001.

Among these five metabolites, cortisone and cortisol both belong to corticosteroid hormones, and cortisone is released by the adrenal gland in response to stress [61]. We observed a positive association between cortisol and hs-CRP and a negative association between CRP and cortisone. One of cortisone’s effects on the body is the suppression of the immune system due to a decrease in the function of the lymphatic system [62], which is consistent with our findings. It was proven in Shimba’s study [63] that corticosteroids are potent inhibitors of inflammatory corneal lymphangiogenesis, with significant differences between various corticosteroids in terms of their antilymphangiogenic potency. The primary mechanism seems to be through the suppression of macrophage infiltration, proinflammatory cytokine expression, and inhibition of the proliferation of lymphatic endothelial cells. A study by Rueggeberg et al. observed that 2-year increases in diurnal cortisol secretion were significantly associated with higher levels of CRP at a 6-year follow-up [64]. These findings may be difficult to reconcile because cortisol generally has anti-inflammatory properties. However, sustained exposure to high cortisol levels may render innate immune cells partially resistant to glucocorticoid inhibition, allowing inflammation to escape normal regulatory controls [65,66]. This could explain the apparent positive correlation between high CRP and cortisol.

The coefficients of cortisol and cortisone show an opposite direction (1.026 vs. −0.91), both being significant. The balance between serum cortisol and cortisone is a known phenomenon, and hence often a ratio is used (cortisol/cortisone), with cortisol being the more active one. Examples of altered rations compared to normal can occur during acute-phase responses [67], in obesity-related issues [68], and in tuberculosis [69]. The relationship in this work may be subject to various factors, including chronic stress, individual differences, and the balance between pro-inflammatory and anti-inflammatory processes in the body. A positive association between cortisol and CRP and a negative association between cortisone and CRP can be explained by the complex and dynamic nature of the body’s stress and inflammatory responses. In some situations, cortisol can have pro-inflammatory effects under chronic stress [70]. Chronic stress can lead to dysregulation of the hypothalamic–pituitary–adrenal axis, resulting in prolonged elevated cortisol levels [68]. These protracted high cortisol levels can induce inflammation and stimulate the production of inflammatory mediators like CRP. A positive association between cortisol and CRP can be observed in this scenario.

Retinol (Vitamin A) exhibited the most robust negative association with hs-CRP among these five notable metabolites in our predictive analysis. In the study by Dios et al. [67], it was investigated that 6–8-year-old children in the highest hs-CRP group (hs-CRP ≥ 0.60 mg/dL) displayed significantly lower levels of retinol compared to those in the lower hs-CRP groups. It is hypothesized that retinol binding to retinol-binding protein (RBP) plays a role in the association between retinol and CRP in children. From a biological perspective, retinol is transported in the blood while bound to RBP, and its concentration is tightly regulated by RBP [68]. Additionally, a correlation between RBP4 and CRP levels has been described in school-aged obese children [71]. Our findings align with previous research and further confirmed the strong correlation between retinol and CRP in a larger, controlled cohort of 6-month-old children. 3Beta-hydroxy-5-cholestenoate (3-BH5C) belongs to the class of monohydroxy bile acids, alcohols, and derivatives. This compound is a component of the primary bile acid biosynthesis pathway and has been recognized as a metabolite capable of predicting gut microbiome Shannon diversity [72]. Shannon diversity serves as a potential marker for overall microbiome health. Our results identified a robust negative association between 3-BH5C and CRP. To the best of our knowledge, this is the first time a negative correlation between 3-BH5C and CRP has been observed, suggesting that the microbiome health of children, as indicated by the marker 3-BH5C, may be linked to inflammation. A prior study conducted in the Northern Finland Birth Cohort 1966 (NFBC1966) and the TwinsUK cohort demonstrated that higher CRP levels were associated with lower alpha diversity of gut microbiome measures [73]. This provides further support for the credibility of our findings.

Another bile acid metabolite, glycocholenate sulfate, classified as a conjugated bile acid (CBA), has been identified as a significant biomarker for CVD risk in a cohort that included 1919 African American participants in the Atherosclerosis Risk study [74]. CBA profiles are increasingly recognized as crucial signaling molecules intricately involved in mammalian cholesterol and lipid metabolism, glucose homeostasis, thermogenesis, inflammation, and intestinal function [75]. Our study further confirmed that CBA, represented by glycocholenate sulfate, is strongly associated with CRP levels in children at six months.

### Limitations and Future Perspective

There are several limitations to consider when interpreting the results. First, the metabolite concentrations were measured semi-quantitatively, which may introduce measurement errors. Secondly, the bioactivity model-based approach used in this study may introduce error propagation and be limited by noise [76] since (a) bioactivity data have a measurement error and (b) the models come with limited accuracy. Furthermore, it is essential to note that the ligand–receptor interactions are complex and may not be fully captured by the model-based approach, a known limitation of QSARs [77]. While efforts were made to incorporate as much information as possible into the models, the true nature of these interactions may be more nuanced than what was captured in this study. There is also no consensus on how to clean data for modeling; hence, the data-cleaning process used in this study may have needed to be more rigorous and lost some information, which is further driven by the unannotated metabolites. Another limitation comes from compounds being excluded in the data processing; however, their effects might be more complex and have a synergy.

A concept worth mentioning is causality, which cannot be assessed using such methods. The exact drivers of metabolic pathways are a question of causality and still need to be completely revealed. Future work should address some of these limitations.

Even though this work is closer to methodological approaches than clinical application, with further validation, it might find its way to the clinics. The hope of metabolomics is, among other things, to support biomarker identification. Such a filtration approach, which can also be perceived as a metabolite selection method, can speed up the identification of biomarkers. Future research might reveal that biomarkers are rather linear and non-linear combinations of metabolites instead of single metabolites. This is also the power of machine learning, which helps generate such combinations in a data-driven manner.

We are also aware that regardless of the children being observed in the clinic and a lot of information being available (see https://copsac.com/home/copsac-cohorts/copsac2010-cohort/, accessed 25 April 2024) that this is only a glimpse of the underlying biology and environmental factors in such processes. In addition to this, both the metabolome and hs-CRP are snapshots and are affected by other processes. More research work is hence needed to understand their variability, as well as single biomarkers and combinations of biomarkers related to physiological conditions.

## 5. Conclusions

We developed a series of QSAR models targeting the Tox21 biological endpoint and used them to predict potential outcomes for each quantified metabolite within the COPSAC2010 cohort. Our analysis unveiled five metabolites of particular interest due to their robust association with hs-CRP levels. We observed the significant influence of CRP on corticosteroid hormones, bile acid metabolites, and vitamin A. The association with corticosteroid hormones is supported by the literature and hence shows this data-driven and chemistry-informed approach to be meaningful, as it leads to known findings without the addition of prior medical knowledge. Our conclusions regarding bile acid metabolites are novel to our understanding. Still, they are supported by the known findings regarding the relationship between microbiome-driven gut health and the level of CRP. Even though causality has not been researched in this paper, we show that there is a relationship between metabolites and CRP. This has the potential to help to understand biological pathways but also for use in clinical settings where supplementation with, e.g., vitamin A and the improvement of gut health might play a role in early childhood.

## Figures and Tables

**Table 1:** Model results for the 12 targets from the Tox21 data set. Models with an asterisk (*) were kept for further processing based on our selection criteria of the MCC CV and MCC Test being > 0.30. The abbreviation BACC is Balanced Accuracy. N(1)/N(0,1) is the imbalance ratio of the positive and total compounds per target.

Endpoint	MCC Train	BACC Train	MCC CV	MCC Test	BACC Test	Num. of Feat.	N(1)/N(0,1)
**SR-HSE**	0.47	0.82	0.27	0.23	0.69	38	340/6402
* NR-AR	0.63	0.95	0.58	0.7	0.97	31	306/7060
* SR-ARE	0.77	0.94	0.35	0.34	0.76	38	945/5758
**NR-Aromatase**	0.6	0.88	0.2	0.15	0.63	38	304/5652
* NR-ER-LBD	0.62	0.89	0.47	0.56	0.84	38	343/6817
* NR-AhR	0.73	0.9	0.51	0.45	0.78	38	756/6459
* SR-MMP	0.83	0.91	0.59	0.58	0.79	38	903/5715
* NR-ER	0.64	0.91	0.41	0.41	0.83	38	806/6056
**NR-PPAR-gamma**	0.59	0.92	0.17	0.09	0.6	13	197/6355
**SR-p53**	0.41	0.63	0.25	0.24	0.58	38	423/6643
* SR-ATAD5	0.52	0.76	0.3	0.31	0.66	38	275/6926
* NR-AR-LBD	0.61	0.81	0.58	0.55	0.82	38	234/6617

**Table 2:** A snippet of probabilities for the first five metabolites being active towards the eight models (columns).

	SR-ATAD5	NR-AR-LBD	NR-AR	NR-ER-LBD	NR-ER	SR-ARE	NR-AhR	SR-MMP
cortisol	0.12	* 0.97	* 0.95	0.07	0.35	0.2	0	0.13
cortisone	0.13	* 0.96	* 0.82	0.04	0.36	0.24	0.02	0.19
androsterone sulfate	0.12	* 0.88	* 0.67	0.43	* 0.59	0.48	0.02	0.33
dehydroepiandrosterone sulfate	0.13	* 0.92	* 0.66	0.46	* 0.67	* 0.54	0.01	0.29
16a-hydroxy dhea 3-sulfate	0.14	* 0.93	* 0.63	0.37	* 0.57	0.42	0.02	0.44

**Table 3:** A list of active metabolites given the pre-trained models.

Active Metabolites	Active Metabolites	Active Metabolites
taurochenodeoxycholic acid 3-sulfate	taurochenodeoxycholate	chenodeoxycholate
4-cholesten-3-one	ursodeoxycholate	2-aminophenol sulfate
gamma-CEHC	pregnenediol sulfate	taurohyocholate
5alpha-pregnan-3beta,20alpha-diol disulfate	16a-hydroxy DHEA 3-sulfate	5alpha-androstan-3beta,17alpha-diol disulfate
cortisone	lithocholate sulfate	taurocholate
taurocholenate sulfate	pregnenetriol sulfate	tauroursodeoxycholate
cholesterol	taurodeoxycholate	glycoursodeoxycholate
alpha-tocopherol	4-hydroxychlorothalonil	N6-methyladenosine
glycodeoxycholate	hyocholate	glycocholate
pregnenolone sulfate	campesterol	3beta-hydroxy-5-cholestenoate
taurolithocholate 3-sulfate	glycochenodeoxycholate	androsterone glucuronide
glycochenodeoxycholate 3-sulfate	isoursodeoxycholate	glycolithocholate
glycolithocholate sulfate	tauro-beta-muricholate	gamma-tocopherol/beta-tocopherol
androsterone sulfate	cortisol	7-HOCA
glycohyocholate	solanidine	beta-cryptoxanthin
caffeine	dehydroepiandrosterone sulfate	piperine
retinol (Vitamin A)	cholate	glycocholenate sulfate
taurochenodeoxycholic acid 3-sulfate	deoxycholate	

**Table 4:** Results of the association testing using linear regression against hs-CRP.

Metabolite	Super_Pathway	Sub_Pathway	coef.	*p*-Value
cortisone	Lipid	Corticosteroids	−0.91	0.000
retinol (Vitamin A)	Cofactors and Vitamins	Vitamin A Metabolism	−1.39	0.000
cortisol	Lipid	Corticosteroids	1.026	0.000
3beta-Hydroxy-5-cholestenoate	Lipid	Sterol (or primary bile acid biosynthesis)	−0.64	0.000
glycocholenate sulfate	Lipid	Secondary Bile Acid Metabolism	0.581	0.002
intercept			2.976	0.000

## Data Availability

All data that support the findings in this study, including clinical data, are available from the corresponding author upon reasonable request: participant-level personally identifiable data are protected under the Danish Data Protection Act and European Regulation 2016/679 of the European Parliament and of the Council (GDPR) that prohibit distribution even in pseudo-anonymized form, but can be made available under a data transfer agreement as a collaboration effort. The data for the QSAR models were taken from the publicly available Tox21 challenge at https://tripod.nih.gov/tox21/challenge/ (access date 25 April 2024).

## Supplementary Materials

The modeling data and auxiliary files are published in (https://zenodo.org/records/10888738, accessed on 25 April 2024).

## References

[1] López-López Á., López-Gonzálvez Á., Barker-Tejeda T.C., Barbas C. (2018). A Review of Validated Biomarkers Obtained through Metabolomics. Expert Rev. Mol. Diagn..

[2] Cardoso M.R., Santos J.C., Ribeiro M.L., Talarico M.C.R., Viana L.R., Derchain S.F.M. (2018). A Metabolomic Approach to Predict Breast Cancer Behavior and Chemotherapy Response. Int. J. Mol. Sci..

[3] Papamichael M.M., Katsardis C., Sarandi E., Georgaki S., Frima E.-S., Varvarigou A., Tsoukalas D. (2021). Application of Metabolomics in Pediatric Asthma: Prediction, Diagnosis and Personalized Treatment. Metabolites.

[4] Gika H., Virgiliou C., Theodoridis G., Plumb R.S., Wilson I.D. (2019). Untargeted LC/MS-Based Metabolic Phenotyping (Metabonomics/Metabolomics): The State of the Art. J. Chromatogr. B.

[5] Rago D., Pedersen C.E.T., Huang M., Kelly R.S., Gürdeniz G., Brustad N., Knihtilä H., Lee-Sarwar K.A., Morin A., Rasmussen M.A. (2021). Characteristics and Mechanisms of a Sphingolipid-Associated Childhood Asthma Endotype. Am. J. Respir. Crit. Care Med..

[6] Johnson C.H., Ivanisevic J., Siuzdak G. (2016). Metabolomics: Beyond Biomarkers and towards Mechanisms. Nat. Rev. Mol. Cell Biol..

[7] Ivanisevic J., Thomas A., Giera M. (2018). Metabolomics as a Tool to Understand Pathophysiological Processes. Clinical Metabolomics: Methods and Protocols.

[8] Nappo A., Iacoviello L., Fraterman A., Gonzalez-Gil E.M., Hadjigeorgiou C., Marild S., Molnar D., Moreno L.A., Peplies J., Sioen I. (2013). High-Sensitivity C-Reactive Protein Is a Predictive Factor of Adiposity in Children: Results of the Identification and Prevention of Dietary- and Lifestyle-Induced Health Effects in Children and Infants (IDEFICS) Study. J. Am. Heart Assoc..

[9] Nishide R., Ando M., Funabashi H., Yoda Y., Nakano M., Shima M. (2015). Association of Serum Hs-CRP and Lipids with Obesity in School Children in a 12-Month Follow-up Study in Japan. Env. Health Prev. Med..

[10] Kumar A., Jat K.R., Sankar J., Lakshmy R., Lodha R., Kabra S.K. (2023). Role of High-Sensitivity C-Reactive Protein (Hs-CRP) in Assessment of Asthma Control in Children. J. Asthma.

[11] Chawes B.L., Stokholm J., Schoos A.-M.M., Fink N.R., Brix S., Bisgaard H. (2017). Allergic Sensitization at School Age Is a Systemic Low-Grade Inflammatory Disorder. Allergy.

[12] Rosenberg J.B., Jepsen J.R.M., Mohammadzadeh P., Sevelsted A., Vinding R., Sørensen M.E., Horner D., Aagaard K., Fagerlund B., Brix S. (2023). Maternal Inflammation during Pregnancy Is Associated with Risk of ADHD in Children at Age 10. Brain Behav. Immun..

[13] Mokkala K., Houttu N., Koivuniemi E., Sørensen N., Nielsen H.B., Laitinen K. (2020). GlycA, a Novel Marker for Low Grade Inflammation, Reflects Gut Microbiome Diversity and Is More Accurate than High Sensitive CRP in Reflecting Metabolomic Profile. Metabolomics.

[14] Cherkasov A., Muratov E.N., Fourches D., Varnek A., Baskin I.I., Cronin M., Dearden J., Gramatica P., Martin Y.C., Todeschini R. (2014). QSAR Modeling: Where Have You Been? Where Are You Going To?. J. Med. Chem..

[15] Gramatica P. (2007). Principles of QSAR Models Validation: Internal and External. QSAR Comb. Sci..

[16] Huang R., Xia M., Nguyen D.-T., Zhao T., Sakamuru S., Zhao J., Shahane S.A., Rossoshek A., Simeonov A. (2016). Tox21Challenge to Build Predictive Models of Nuclear Receptor and Stress Response Pathways as Mediated by Exposure to Environmental Chemicals and Drugs. Front. Environ. Sci..

[17] Richard A.M., Huang R., Waidyanatha S., Shinn P., Collins B.J., Thillainadarajah I., Grulke C.M., Williams A.J., Lougee R.R., Judson R.S. (2020). The Tox21 10K Compound Library: Collaborative Chemistry Advancing Toxicology. Chem. Res. Toxicol..

[18] Heinze K., Lin A., Reniers R.L.E.P., Wood S.J. (2016). Longer-Term Increased Cortisol Levels in Young People with Mental Health Problems. Psychiatry Res..

[19] Joseph J.J., Golden S.H. (2017). Cortisol Dysregulation: The Bidirectional Link between Stress, Depression, and Type 2 Diabetes Mellitus. Ann. N. Y. Acad. Sci..

[20] van Spronsen F.J., Hoeksma M., Reijngoud D.-J. (2009). Brain Dysfunction in Phenylketonuria: Is Phenylalanine Toxicity the Only Possible Cause?. J. Inherit. Metab. Dis..

[21] Lee N., Spears M.E., Carlisle A.E., Kim D. (2020). Endogenous Toxic Metabolites and Implications in Cancer Therapy. Oncogene.

[22] Chawes B.L., Bønnelykke K., Stokholm J., Vissing N.H., Bjarnadóttir E., Schoos A.-M.M., Wolsk H.M., Pedersen T.M., Vinding R.K., Thorsteinsdóttir S. (2016). Effect of Vitamin D3 Supplementation During Pregnancy on Risk of Persistent Wheeze in the Offspring: A Randomized Clinical Trial. JAMA.

[23] Bisgaard H., Stokholm J., Chawes B.L., Vissing N.H., Bjarnadóttir E., Schoos A.-M.M., Wolsk H.M., Pedersen T.M., Vinding R.K., Thorsteinsdóttir S. (2016). Fish Oil–Derived Fatty Acids in Pregnancy and Wheeze and Asthma in Offspring. N. Engl. J. Med..

[24] Gürdeniz G., Ernst M., Rago D., Kim M., Courraud J., Stokholm J., Bønnelykke K., Björkbom A., Trivedi U., Sørensen S.J. (2022). Neonatal Metabolome of Caesarean Section and Risk of Childhood Asthma. Eur. Respir. J..

[25] Rago D., Rasmussen M.A., Lee-Sarwar K.A., Weiss S.T., Lasky-Su J., Stokholm J., Bønnelykke K., Chawes B.L., Bisgaard H. (2019). Fish-Oil Supplementation in Pregnancy, Child Metabolomics and Asthma Risk. eBioMedicine.

[26] Sumner L.W., Amberg A., Barrett D., Beale M.H., Beger R., Daykin C.A., Fan T.W.-M., Fiehn O., Goodacre R., Griffin J.L. (2007). Proposed Minimum Reporting Standards for Chemical Analysis. Metabolomics.

[27] Olarini A., Ernst M., Gürdeniz G., Kim M., Brustad N., Bønnelykke K., Cohen A., Hougaard D., Lasky-Su J., Bisgaard H. (2022). Vertical Transfer of Metabolites Detectable from Newborn’s Dried Blood Spot Samples Using UPLC-MS: A Chemometric Study. Metabolites.

[28] Fink N.R., Chawes B., Bønnelykke K., Thorsen J., Stokholm J., Rasmussen M.A., Brix S., Bisgaard H. (2019). Levels of Systemic Low-Grade Inflammation in Pregnant Mothers and Their Offspring Are Correlated. Sci. Rep..

[29] Armitage E.G., Godzien J., Alonso-Herranz V., López-Gonzálvez Á., Barbas C. (2015). Missing Value Imputation Strategies for Metabolomics Data. Electrophoresis.

[30] Lovrić M., Horner D., Chen L., Brustad N., Malby Schoos A.-M., Lasky-Su J., Chawes B., Rasmussen M.A. (2024). Vertical Metabolome Transfer from Mother to Child: An Explainable Machine Learning Method for Detecting Metabolomic Heritability. Metabolites.

[31] Fernandez M., Ban F., Woo G., Hsing M., Yamazaki T., Leblanc E., Rennie P.S., Welch W.J., Cherkasov A. (2018). Toxic Colors: The Use of Deep Learning for Predicting Toxicity of Compounds Merely from Their Graphic Images. J. Chem. Inf. Model..

[32] Hemmerich J., Asilar E., Ecker G.F. (2019). Conformational Oversampling as Data Augmentation for Molecules. Lecture Notes in Computer Science.

[33] Idakwo G., Thangapandian S., Luttrell J., Li Y., Wang N., Zhou Z., Hong H., Yang B., Zhang C., Gong P. (2020). Structure–Activity Relationship-Based Chemical Classification of Highly Imbalanced Tox21 Datasets. J. Cheminformatics.

[34] Mayr A., Klambauer G., Unterthiner T., Hochreiter S. (2016). DeepTox: Toxicity Prediction Using Deep Learning. Front. Environ. Sci..

[35] Klimenko K., Rosenberg S.A., Dybdahl M., Wedebye E.B., Nikolov N.G. (2019). QSAR Modelling of a Large Imbalanced Aryl Hydrocarbon Activation Dataset by Rational and Random Sampling and Screening of 80,086 REACH Pre-Registered and/or Registered Substances. PLoS ONE.

[36] Lovrić M., Đuričić T., Tran H.T.N., Hussain H., Lacić E., Rasmussen M.A., Kern R. (2021). Should We Embed in Chemistry? A Comparison of Unsupervised Transfer Learning with PCA, UMAP, and VAE on Molecular Fingerprints. Pharmaceuticals.

[37] Lovrić M., Molero J.M., Kern R. (2019). PySpark and RDKit: Moving towards Big Data in Cheminformatics. Mol. Inform..

[38] Fourches D., Muratov E., Tropsha A. (2010). Trust, but Verify: On the Importance of Chemical Structure Curation in Cheminformatics and QSAR Modeling Research. J. Chem. Inf. Model..

[39] Rogers D., Hahn M. (2010). Extended-Connectivity Fingerprints. J. Chem. Inf. Model..

[40] Landrum G. RDKit: Open-Source Cheminformatics Software. http://rdkit.org/.

[41] Gütlein M., Kramer S. (2016). Filtered Circular Fingerprints Improve Either Prediction or Runtime Performance While Retaining Interpretability. J. Cheminformatics.

[42] Landrum G. RDKit: Colliding Bits III. http://rdkit.blogspot.com/2016/02/colliding-bits-iii.html.

[43] Lovrić M., Pavlović K., Žuvela P., Spataru A., Lučić B., Kern R., Wong M.W. (2021). Machine Learning in Prediction of Intrinsic Aqueous Solubility of Drug-like Compounds: Generalization, Complexity, or Predictive Ability?. J. Chemom..

[44] Pedregosa F., Michel V., Grisel O., Blondel M., Prettenhofer P., Weiss R., Vanderplas J., Cournapeau D., Varoquaux G., Gramfort A. (2011). Scikit-Learn: Machine Learning in Python. J. Mach. Learn. Res..

[45] Boughorbel S., Jarray F., El-Anbari M. (2017). Optimal Classifier for Imbalanced Data Using Matthews Correlation Coefficient Metric. PLoS ONE.

[46] Lovrić M., Malev O., Klobučar G., Kern R., Liu J.J., Lučić B. (2021). Predictive Capability of QSAR Models Based on the CompTox Zebrafish Embryo Assays: An Imbalanced Classification Problem. Molecules.

[47] Lučić B., Batista J., Bojović V., Lovrić M., Sović Kržić A., Bešlo D., Nadramija D., Vikić-Topić D. (2019). Estimation of Random Accuracy and Its Use in Validation of Predictive Quality of Classification Models within Predictive Challenges. Croat. Chem. Acta.

[48] Snoek J., Larochelle H., Adams R.P. Practical Bayesian Optimization of Machine Learning Algorithms. Proceedings of the NIPS 2012.

[49] Ginebreda A., Kuzmanovic M., Guasch H., de Alda M.L., López-Doval J.C., Muñoz I., Ricart M., Romaní A.M., Sabater S., Barceló D. (2014). Assessment of Multi-Chemical Pollution in Aquatic Ecosystems Using Toxic Units: Compound Prioritization, Mixture Characterization and Relationships with Biological Descriptors. Sci. Total Environ..

[50] Vallat R. (2018). Pingouin: Statistics in Python. JOSS.

[51] Lovrić M. Modelling Data and Model Results Tox21 (0.1) [Data Set]. Zenodo. https://zenodo.org/records/10888738.

[52] Plebani M. (2023). Why C-Reactive Protein Is One of the Most Requested Tests in Clinical Laboratories?. Clin. Chem. Lab. Med..

[53] Windgassen E.B., Funtowicz L., Lunsford T.N., Harris L.A., Mulvagh S.L. (2011). C-Reactive Protein and High-Sensitivity C-Reactive Protein: An Update for Clinicians. Postgrad. Med..

[54] Fonseca F.A.H., de Oliveira Izar M.C. (2016). High-Sensitivity C-Reactive Protein and Cardiovascular Disease Across Countries and Ethnicities. Clinics.

[55] Hod K., Ringel-Kulka T., Martin C.F., Maharshak N., Ringel Y. (2016). High-Sensitive C-Reactive Protein as a Marker for Inflammation in Irritable Bowel Syndrome. J. Clin. Gastroenterol..

[56] Valkanova V., Ebmeier K.P., Allan C.L. (2013). CRP, IL-6 and Depression: A Systematic Review and Meta-Analysis of Longitudinal Studies. J. Affect. Disord..

[57] Chawes B.L.K., Stokholm J., Bønnelykke K., Brix S., Bisgaard H. (2015). Neonates with Reduced Neonatal Lung Function Have Systemic Low-Grade Inflammation. J. Allergy Clin. Immunol..

[58] Rahman Fink N., Chawes B.L., Thorsen J., Stokholm J., Krogfelt K.A., Schjørring S., Kragh M., Bønnelykke K., Brix S., Bisgaard H. (2018). Neonates Colonized with Pathogenic Bacteria in the Airways Have a Low-Grade Systemic Inflammation. Allergy.

[59] Shimoda T., Obase Y., Kishikawa R., Iwanaga T. (2015). Serum High-Sensitivity C-Reactive Protein Can Be an Airway Inflammation Predictor in Bronchial Asthma. Allergy Asthma Proc..

[60] Kurosaki K., Wu R., Uesawa Y. (2020). A Toxicity Prediction Tool for Potential Agonist/Antagonist Activities in Molecular Initiating Events Based on Chemical Structures. Int. J. Mol. Sci..

[61] Nicolaides N.C., Kyratzi E., Lamprokostopoulou A., Chrousos G.P., Charmandari E. (2014). Stress, the Stress System and the Role of Glucocorticoids. Neuroimmunomodulation.

[62] Hos D., Saban D.R., Bock F., Regenfuss B., Onderka J., Masli S., Cursiefen C. (2011). Suppression of Inflammatory Corneal Lymphangiogenesis by Application of Topical Corticosteroids. Arch. Ophthalmol..

[63] Shimba A., Ikuta K. (2020). Control of Immunity by Glucocorticoids in Health and Disease. Semin. Immunopathol..

[64] Rueggeberg R., Wrosch C., Miller G.E., McDade T.W. (2012). Associations between Health-Related Self-Protection, Diurnal Cortisol, and C-Reactive Protein in Lonely Older Adults. Psychosom. Med..

[65] Miller G.E., Chen E., Sze J., Marin T., Arevalo J.M.G., Doll R., Ma R., Cole S.W. (2008). A Functional Genomic Fingerprint of Chronic Stress in Humans: Blunted Glucocorticoid and Increased NF-κB Signaling. Biol. Psychiatry.

[66] Raison C.L., Miller A.H. (2011). When Not Enough Is Too Much: The Role of Insufficient Glucocorticoid Signaling in the Pathophysiology of Stress-Related Disorders. FOC.

[67] De Dios O., Navarro P., Ortega-Senovilla H., Herrero L., Gavela-Pérez T., Soriano-Guillen L., Lasunción M.A., Garcés C. (2018). Plasma Retinol Levels and High-Sensitivity C-Reactive Protein in Prepubertal Children. Nutrients.

[68] García O.P., Ronquillo D., Del Carmen Caamaño M., Martínez G., Camacho M., López V., Rosado J.L. (2013). Zinc, Iron and Vitamins A, C and E Are Associated with Obesity, Inflammation, Lipid Profile and Insulin Resistance in Mexican School-Aged Children. Nutrients.

[69] Baker R.W., Walker B.R., Shaw R.J., Honour J.W., Jessop D.S., Lightman S.L., Zumla A., Rook G.A. (2000). Increased Cortisol: Cortisone Ratio in Acute Pulmonary Tuberculosis. Am. J. Respir. Crit. Care Med..

[70] Cruz-Topete D., Cidlowski J.A. (2015). One Hormone, Two Actions: Anti- and pro-Inflammatory Effects of Glucocorticoids. Neuroimmunomodulation.

[71] Balagopal P., Graham T.E., Kahn B.B., Altomare A., Funanage V., George D. (2007). Reduction of Elevated Serum Retinol Binding Protein in Obese Children by Lifestyle Intervention: Association with Subclinical Inflammation. J. Clin. Endocrinol. Metab..

[72] Wilmanski T., Rappaport N., Earls J.C., Magis A.T., Manor O., Lovejoy J., Omenn G.S., Hood L., Gibbons S.M., Price N.D. (2019). Blood Metabolome Predicts Gut Microbiome α-Diversity in Humans. Nat. Biotechnol..

[73] Zouiouich S., Loftfield E., Huybrechts I., Viallon V., Louca P., Vogtmann E., Wells P.M., Steves C.J., Herzig K.-H., Menni C. (2021). Markers of Metabolic Health and Gut Microbiome Diversity: Findings from Two Population-Based Cohort Studies. Diabetologia.

[74] Torres-Barrán A., Alonso Á., Dorronsoro J.R. (2019). Regression Tree Ensembles for Wind Energy and Solar Radiation Prediction. Neurocomputing.

[75] Fava F., Rizzetto L., Tuohy K.M. (2019). Gut Microbiota and Health: Connecting Actors across the Metabolic System. Proc. Nutr. Soc..

[76] Kolmar S.S., Grulke C.M. (2021). The Effect of Noise on the Predictive Limit of QSAR Models. J. Cheminformatics.

[77] Myshkin E., Brennan R., Khasanova T., Sitnik T., Serebriyskaya T., Litvinova E., Guryanov A., Nikolsky Y., Nikolskaya T., Bureeva S. (2012). Prediction of Organ Toxicity Endpoints by QSAR Modeling Based on Precise Chemical-Histopathology Annotations. Chem. Biol. Drug Des..

